# The potential of new prediction models for emergency medical dispatch prioritisation of patients with chest pain: a cohort study

**DOI:** 10.1186/s13049-022-01021-5

**Published:** 2022-05-08

**Authors:** Kristoffer Wibring, Markus Lingman, Johan Herlitz, Angela Bång

**Affiliations:** 1 Department of Ambulance and Prehospital Care, Region Halland, Varlabergsvägen 29, 434 39 Kungsbacka, Sweden; 2 Institute of Health and Care Sciences, Sahlgrenska Academy, University of Gothenburg, Gothenburg, Sweden; 3 Department of Molecular and Clinical Medicine/Cardiology, Institute of Medicine, Sahlgrenska Academy, University of Gothenburg, Gothenburg, Sweden; 4 Department of Development, Halland Hospital, Halmstad, Sweden; 5 The Prehospital Research Center Western Sweden, University of Borås, Borås, Sweden

**Keywords:** Chest pain, Emergency medical services, Emergency medical dispatch, Acute coronary syndrome, Signs and symptoms, Risk assessment, Prioritisation

## Abstract

**Objectives:**

To develop emergency medical dispatch (EMD) centre prediction models with high sensitivity and satisfying specificity to identify high-priority patients and patients suitable for non-emergency care respectively, when assessing patients with chest pain.

**Methods:**

Observational cohort study of 2917 unselected patients with chest pain who contacted an EMD centre in Sweden due to chest pain during 2018. Multivariate logistic regression was applied to develop models predicting low-risk or high-risk condition, that is, occurrence of time-sensitive diagnosis on hospital discharge.

**Results:**

Prediction models were developed for the identification of patients suitable for high- and low-priority dispatch, using 11 and 10 variables respectively. The area under the receiver-operating characteristic curve (AUROC) for the high-risk prediction model was 0.79 and for the low-risk model it was 0.74. When applying the high-risk prediction model, 56% of the EMS missions were given highest priority, compared with 65% with the current standard. When applying the low-risk model, 7% were given the lowest priority compared to 1% for the current standard. The new prediction models outperformed today’s dispatch priority accuracy in terms of sensitivity as well as positive and negative predictive value in both high- and low-risk prediction. The low-risk model predicted almost six times as many patients as having low-risk conditions compared with today’s standard. This was done without increasing the number of high-risk patients wrongly assessed as low-risk.

**Conclusions:**

By introducing prediction models, based on logistic regression analyses, using variables obtained by standard EMD-questions on age, sex, medical history and symptomology, EMD prioritisation can be improved compared with using current criteria index-based ones. This will allow a more efficient emergency medical services resource allocation.

## Background

Chest pain is one of the most common reasons for calling the emergency medical dispatch (EMD) centre, accounting for more than ten percent of all calls [1]. Chest pain can be caused by several high-risk conditions where time is crucial, such as myocardial infarction, aortic aneurysm and pulmonary embolism. For this reason, high sensitivity is warranted for EMD assessment of patients with chest pain. However, in most cases chest pain is benign [2]. The desire to attain high sensitivity and the relative rareness of high-risk conditions give rise to substantial over-triage, giving patients with low-risk conditions the highest dispatch priority [2, 3]. In addition, the proportion of emergency medical services (EMS) patients in whom chest pain is caused by acute myocardial infarction (AMI) has decreased during the last decades [2, 4, 5]. During the same time period, the overall number of EMS requests has increased [1, 6, 7] altering EMD call probabilities. The increased utilisation of the EMS may partly be explained by numerous information campaigns to the public encouraging them to call the EMD when experiencing chest pain [8–11]. However, both EMS utilisation and EMD prioritisation are complex and affected by several factors, such as patients’ socio-economic background. For example, high socio-economic status shortens EMS response times for patients with chest pain [12–14]. Furthermore, EMS utilisation is higher in low-income areas [15, 16] while a smaller proportion are given highest priority by the EMD [17] or receive advanced prehospital care [12].

A displacement effect results from the combination of four factors: a general increase in EMS utilisation [1, 6, 7]; chest pain being a common cause for EMD contact [1]; a decreasing proportion of patients with chest pain from AMI [2, 4, 5]; and substantial over-triage of patients with chest pain [2, 5]. This means that patients with non-urgent healthcare needs are given high priority and occupy limited EMS resources at the cost of delaying EMS care for those with more urgent needs. This displacement effect can be compared with the well-known phenomenon of emergency department crowding, when an increase in patient volume negatively affects the quality and promptness of care provided, leading to an increase in adverse events [18–20].

For cardiac arrest, one of the most urgent conditions in EMS care, EMS response times in Sweden have doubled during the last decades [21]. This may probably be explained at least partly by the displacement effect described above. Estimations claim that lowering the cardiac arrest response times to the 1990 level would double the survival rates [21].

The increasing number of EMD contacts due to chest pain, the advice to the public to contact the EMD when experiencing chest pain, and the decreasing proportion of high-risk conditions all emphasise the need for more accurate assessment and prioritisation at the EMD. Improving the accuracy of EMD prioritisation of patients with chest pain would lead to more patients with high-risk conditions having an ambulance dispatched with high priority and fewer patients with low-risk conditions being given high priority, thereby reducing over-triage. This would result in more effective EMS resource coordination and influence the displacement effect positively. Identifying patients with chest pain suitable for other modes of transportation than ambulance would also influence the displacement effect positively.

The aim of this study has been to develop an EMD prediction model with high sensitivity and satisfying specificity to identify high-priority patients and patients suitable for non-emergency care when assessing patients with chest pain.

## Methods

This study is part of the BRIAN research programme (BRöstsmärta I AmbulaNs (Swedish), EMS Chest Pain (English)). The cohort, methods and previous results have been described elsewhere [22, 23] and are therefore summarised briefly.

### Setting

In Sweden, 112 is the emergency number used by all EMD centres. In the county studied, the EMD centre is run by the company SOS Alarm AB, owned by the Swedish Government and the Swedish Association of Local Authorities and Regions. At the EMD centre, the emergency medical call is assessed by an emergency medical dispatch operator. The EMD operator does not need any formal medical education but must have undergone six months of training and certification before starting to handle emergency medical calls single-handed. The EMD operator uses the “Swedish Index for Medical Priority Dispatch” (SIMPD) when setting the priority of the EMS mission. The SIMPD is a criteria-based index adapted from a Norwegian version that was developed from the Medical Priority Dispatch System (MPDS) used worldwide [24–26]. The SIMPD has just over 30 chapters of which chest pain is one. The EMD operator uses the SIMPD to assign a priority level from 1 (the most urgent) to 3 (least urgent) [27]. The patient may also be referred to waiting at home, other modes of transportation than ambulance or contacting a non-emergency care agency for medical consultation such as a primary healthcare centre [24]. If the operator is in doubt when assessing an emergency medical call, a registered nurse may be consulted [24, 27].

The county studied covers an area of 5500 km^2^ and had 329,000 inhabitants in 2018. These are served by two emergency hospitals. The EMS consist of eight ambulance stations with 19 ambulances during daytime. This ambulance fleet is coordinated by an EMD operator who determines which ambulance should be allocated to which EMS mission. In 2018, approximately 30,000 patient-related EMS missions were carried out (inter-hospital site transports excluded).

### Study population

A total of 3121 EMS missions were carried out in the county catchment area including patients ≥ 18 years old and with a chief complaint of chest pain (assigned Rapid Emergency Triage and Treatment System (RETTS) code 5, i.e. chest pain, according to EMS personnel on scene [28]). All these missions were eligible for inclusion. After excluding patients declining to participate and patients who were lost to follow-up, 2917 EMS missions remained and were consecutively included in the study.

### Data collection

Each patient was tracked throughout the emergency healthcare chain, from EMD prioritisation to hospital discharge. Data on symptoms was retrieved using a 15-item questionnaire [22] embedded in the digital EMS record available bedside. Previous medical history and diagnosis on hospital discharge were collected from the hospital medical records. Thus data on symptoms and previous medical history was not collected during the emergency medical call, but later in the healthcare chain.

### Endpoint

The primary endpoint was a risk classification group, in terms of a low- or high-risk condition. All patients were classified as having either a low-, intermediate- or high-risk condition as the cause of their chest pain. The assessment was based on the final diagnosis on discharge from hospital according to the physician in charge. A high-risk condition was defined as a time-sensitive condition with high risk of death and in need of immediate hospital care. An intermediate-risk condition was defined as a condition probably in need of hospital care, but for which time was not a critical factor. A low-risk condition was defined as a final diagnosis with no medical need for prompt hospital care. An overview of how the different diagnoses were classified along with a more detailed description of how this risk classification was carried out can be found in a previous publication [22]. In the EMD setting this could be translated to:

- High-risk condition—in need of highest dispatch priority.
- Intermediate-risk condition—ambulance dispatch with a lower priority is appropriate.
- Low-risk condition—no need for ambulance dispatch, referral to alternative modes of transportation or contacting a non-emergency care agency are appropriate.

### Data imputation

The general idea is iteratively to train a machine learning model on complete variables to predict unknown instances of any missing variable. The list of complete variables is updated at every iteration by including the newly imputed variable, thereby using better and better data for every iteration. We leveraged the MissForest algorithm that iteratively builds Random Forest models to impute missing instances in the variables of interest [29], to improve the prerequisites for multivariate analysis. Random Forest combines the predictions from several regression trees to produce more accurate predictions and bootstrapping to reduce the risk of overfitting [30].

### Statistical analysis

After imputation, the complete data set of 2917 EMS missions was randomly divided into two sets [31]: one prediction model training set including 2049 (70%) EMS missions and one validation set including 868 (30%) EMS missions. Two prediction models were generated from the training set, one for low- and one for high-risk prediction.

Stepwise forward logistic regression was used when generating the prediction models. To limit the number of variables in the models, thereby easing clinical usage and strengthening generalisability, strict thresholds for variable entry and exit were applied (*p* ≤ 0.001 for both).

For the high-risk model, a low (10%) endpoint probability cut-off was set to ensure high sensitivity, thereby reducing the risk for wrongly down-prioritising patients with high-risk conditions. For the low-risk model, a high (90%) endpoint probability cut-off was set to ensure high specificity, thereby reducing the risk of assessing patients with high-risk conditions as suitable for non-ambulance dispatch.

McNemar’s test [32, 33] was used to analyse possible differences between SIMPD/EMD prioritisation and prioritisation based on the prediction models developed.

All statistical analyses were carried out using IBM SPSS Statistics 27. Imputation was implemented using Python (Scikit-learn package).

## Results

Of the 868 EMS missions included in the validation set, 17% concerned patients with high-risk conditions. The corresponding figures for intermediate-risk and low-risk conditions were 15% and 68% respectively. The median age was 72 years old (Q25–Q75, 58–82), and 50% were men (Table 1). This is equivalent to both the complete set described in detail in the previous report [22] and the training set used for prediction model development in this study, which indicates successful randomisation.

**Table 1:** Description of complete, training and validation set

	Complete set, n (%)	Training set, n (%)	Validation set, n (%)
All	2917 (100)	2049 (100)	868 (100)
Male sex	1465 (50)	1035 (51)	430 (50)
Median age (Q1–Q3)	72 (58–82)	72 (58–82)	72 (52–82)
High-risk condition	467 (16)	318 (16)	149 (17)
Intermediate-risk condition	455 (16)	328 (16)	127 (15)
Low-risk condition	1995 (68)	1403 (68)	592 (68)

The stepwise forward logistic regression generated a high-risk condition prediction model with 11 different variables. The variables most indicative of a high-risk condition were pain during activity, intense pain and pain in right arm (Table 2).

**Table 2:** Prediction model for high-risk conditions (missions to assign high priority)

	*p*-value^*^	Odds ratio	Confidence interval, 99.9%	
			Lower	Upper
Age	< 0.001	1.04	1.02	1.05
Male sex	< 0.001	1.89	1.21	2.96
Previous history of COPD	< 0.001	0.38	0.16	0.92
Previous history of atrial fibrillation	< 0.001	0.29	0.16	0.53
Pale	< 0.001	2.37	1.38	4.06
NRS > 8	< 0.001	2.69	1.09	6.64
Pain debut during activity	< 0.001	2.48	1.46	4.18
Constant pain	< 0.001	1.84	1.15	2.94
Pain in right arm	< 0.001	3.07	1.38	6.79
Pressuring pain	< 0.001	1.75	1.04	2.95
Central chest pain	< 0.001	1.70	1.05	2.75

*COPD* chronic obstructive pulmonary disease, *NRS*  numeric rating scale

*Stepwise forward logistic regression, p-value for entry ≤ 0.001 and for exit ≤ 0.001

The stepwise forward logistic regression generated a low-risk condition prediction model with 10 different variables. Most variables had an odds ratio of less than one, thereby signifying reduced chance of a low-risk condition. The variables with increased odds ratios for a low-risk condition were previous history of angina pectoris and sudden pain debut (within seconds) (Table 3). The models for high- and low-risk prediction respectively shared six variables. In total, if applying both models at the same time, 15 different variables are included.

**Table 3:** Prediction model for low-risk conditions (missions without need of EMS care)

	*p*-value*	Odds ratio	Confidence interval, 99.9%	
			Lower	Upper
Age	< 0.001	0.97	0.96	0.98
Male sex	< 0.001	0.58	0.41	0.82
Previous history of angina pectoris	< 0.001	1.92	1.22	3.00
Pale	< 0.001	0.36	0.23	0.58
Patient experiencing affected breathing		0.65	0.46	0.93
Sudden pain debut, within seconds	< 0.001	1.48	1.01	2.18
Constant pain	0.001	0.62	0.43	0.89
Pain in right arm	< 0.001	0.45	0.22	0.92
Pressuring pain	< 0.001	0.59	0.40	0.86
Left-sided chest pain	< 0.001	1.80	1.19	2.71

*COPD* chronic obstructive pulmonary disease, *NRS*  numeric rating scale

*Stepwise forward logistic regression, *p*-value for entry ≤ 0.001 and for exit ≤ 0.001

The area under the receiver-operating characteristic curve (AUROC) for the high-risk model was 0.79 when applied to the validation set. For the low-risk prediction model, the AUROC was 0.74 (Table 4).

**Table 4:** Prediction model accuracy (validation set)

	High-risk prediction		Low-risk prediction	
	Prediction model	Swedish index for medical priority dispatch	Prediction model	Swedish index for medical priority dispatch
Number of variables in model	11	–	10	–
Cut-off, %	10	–	90	–
Sensitivity, %	89	77	10	1
Specificity, %	51	38	97	99
Positive predictive value, %	27	20	89	64
Negative predictive value, %	96	89	33	32
AUROC	0.789	–	0.737	–
Highest priority dispatch, n (%)	488 (56)	563 (65)	–	–
Lowest priority dispatch, n (%)	–	–	64 (7)	11 (1)
Patients with high-risk condition given lowest priority, n (%)			3 (5)	3 (27)

*AUROC* area under receiver operating characteristic curve

Calculations based on validation set with 868 EMS missions

In the validation set, 65% of the EMS missions were given highest priority by the EMD operator when using the SIMPD. The corresponding figure for lowest priority was 1%. When the high-risk prediction model was applied instead, 56% of the EMS missions were given highest priority. When the low-risk model was applied, 7% were given the lowest priority. The new prediction models outperformed the SIMPD regarding sensitivity as well as positive and negative predictive value for both high- and low-risk prediction. The SIMPD was slightly better than the new prediction models concerning specificity when identifying patients with low-risk conditions. When comparing the models developed here with the SIMPD, using McNemar’s test, both the high- and low-risk differed significantly from the SIMPD (*p*-value < 0.001).

Three patients with high-risk conditions were wrongly assessed by both the SIMPD and the low-risk prediction model as having low-risk conditions. However, the low-risk model predicted almost six times as many patients as having low-risk conditions, and thereby displayed a lower misclassification rate. The three high-risk patients wrongly classified by the low-risk prediction model suffered from non-ST-elevation myocardial infarction (NSTEMI), pulmonary embolism, and sepsis respectively. Two of the high-risk patients wrongly assigned lowest priority by the SIMPD suffered from NSTEMI and one from unstable angina.

## Discussion

In this study, we show that prediction models based on ten and eleven variables respectively outperform the decision support tool (SIMPD) currently used. They outperform both in the identification of patients needing an ambulance with highest priority and patients with less urgent medical needs who could be referred to transport/care other than EMS transport to the emergency department.

The prediction model variables included can be obtained by the EMD operator by asking questions which must be considered standard when evaluating patients with chest pain: *“Is your breathing affected?” “Can you describe the character of your pain?” “What part of your chest is hurting?”* etc. along with standard EMD information regarding age, sex and medical history. Such questions could be integrated into a computer-aided dispatch system and automatically supplied to the EMD operator when chest pain is selected as the chief complaint. Rawshani et al. [34] have previously shown that such an approach is feasible when EMD operators are instructed to ask ten questions regarding symptoms and previous medical history when handling calls concerning patients with chest pain. If such questions are asked and the answers obtained are entered onto a computer to allow the prediction model to estimate the likelihood of a high- or low-risk condition, the results of that estimation can support the EMD operator when assessing the appropriate priority.

This study shows that the accuracy of the SIMPD currently used is quite modest in terms of identifying patients with high-risk or low-risk conditions. By applying the newly developed prediction models, accuracy can be improved and still decrease the number of high priority dispatches. Thus more patients with urgent care needs are given high priority and at the same time sparse EMS resources are used more effectively. Even if the accuracy of the newly developed prediction models is not perfect, they outperform the current standard model.

The misclassification rate was 5% for patients predicted as having low-risk conditions but actually having high-risk conditions. Thus it does not seem feasible for the EMD operator to refer patients with chest pain to primary care instead of the emergency department, and still provide acceptable patient safety [35, 36]. This is also in line with previously developed prediction models [37]. If an ambulance with lowest priority or a single-manned unit is instead dispatched to patients predicted as having low-risk conditions, patient safety can be maintained while still allowing more effective EMS resource utilisation compared with the SIMPD.

Gellerstedt et al. [5] developed a prediction model to be used at the EMD centre to identify patients with life-threatening conditions among patients with chest pain. This model had a sensitivity of 88% which is equivalent to the high-risk model developed in this study. The corresponding figure for the SIMPD is 77%, the same as in our study. The results of the Gellerstedt et al. [5] study support our finding that computer-based prediction models based on logistic regression analyses outperform a criteria-based index such as the SIMPD regarding the identification of high-risk patients, without increasing the dispatch of EMS resources with highest priority. It therefore seems reasonable to advocate the introduction of more advanced and dynamic decision support systems at the EMD centre than the current criteria-based ones.

### Strengths and limitations

A key strength is the unselected inclusion of EMS missions comprising an almost complete population of EMS chest pain patients for one year, which improves generalisability. Gellerstedt et al. [5] report comparable data on demographics and priority distribution for their cohort which further strengthens the external validity of our findings.

Another strength is the use of two different sets for model training and validation. This increases the generalisability of the results, and also makes it more likely that the newly developed prediction models will also perform well in other cohorts. However, external validation using an out-of-sample data set is needed before considering clinical implementation.

One weakness of this study is that it is based on data collected by EMS personnel on scene but not during emergency medical calls. It is possible that data collected by the EMD might differ in terms of pain intensity for example, or assessment of paleness. This also makes it difficult to assess the practicability of assigning the EMD operator to obtain the data required for the prediction models. Since this data can be obtained by asking conventional questions on demographics and symptomology, we judge that in most cases this would not entail any problems, which is also supported by Rawshani et al. [34].

Furthermore, the use of non-EMD data did not allow us to investigate whether obtaining the data would extend the length of the emergency medical call. EMD centre calls concerning chest pain patients with high-risk conditions, when using the SIMPD, last for a median time of 4 min [38], and median time from dispatch to EMS arrival is 10 min in the county studied [22]. A possible extension of call length by a few percent is thus unlikely to affect patient outcome, especially since most EMS chest pain dispatches concern patients with low-risk conditions. One should also be aware that it is common practice in some cases to dispatch an ambulance on information retrieved early in the emergency medical call and then reclassify the priority, or even cancel the EMS mission, as the call progress continues and further questions are asked. This limits the delay of EMS response times due to long-lasting emergency medical calls. On the other hand, this approach may complicate the use of the prediction models developed as they require complete data to provide a prediction. However, it is reasonable to assume that this potential delay would be compensated for by the more optimised resource allocation enabled by more accurate assessments and prioritisations.

The results regarding the accuracy of the SIMPD are probably not directly transferable to other EMD support systems such as the MPDS, from which the SIMPD was developed some decades ago [24]. The SIMPD and the MPDS have evolved independently of each other for several years. However, the design and structure of the SIMPD and the MPDS remain the same and the potential benefits of applying prediction models such as the one developed in this study probably also apply to the MPDS.

## Conclusions

We conclude that by introducing prediction models based on logistic regression analyses, using variables obtained by standard EMD questions on age, sex, medical history and symptomology, EMD prioritisation can be improved compared with using current criteria index-based ones. This would allow more efficient EMS resource allocation. We advocate external validation of available computer-based prediction models to allow implementation at the EMD centre, to support the operators in their decision-making.

## Data Availability

The datasets generated and analysed during the current study are not publicly available due the integrity of patient privacy but are available from the corresponding author on reasonable request and if approved by the Regional Ethical Review Board in Lund.

## Abbreviations

AMI: Acute Myocardial Infarction
AUROC: Area Under the Receiver-Operating Characteristic Curve
EMD: Emergency Medical Dispatch
EMS: Emergency Medical Services
MPDS: Medical Priority Dispatch System
NSTEMI: Non-ST-elevation myocardial infarction
SIMPD: Swedish Index for Medical Priority Dispatch
